# Effect of copper, arsenic and nickel on pyrite-based autotrophic denitrification

**DOI:** 10.1007/s10532-023-10027-4

**Published:** 2023-04-28

**Authors:** Maria F. Carboni, Sonia Arriaga, Piet N. L. Lens

**Affiliations:** 1 National University of Ireland Galway, University Road, Galway, H91 TK33 Ireland; 2 División de Ciencias Ambientales, Instituto Potosino de Investigación Científica y Tecnológica, Camino a la Presa San José 2055, Lomas 4a Sección, 78216 San Luis Potosí, CP Mexico; 3 School of Natural Science and Ryan Institute, National University of Ireland Galway, University Road, Galway, H91 TK33 Ireland

**Keywords:** Nitrate, Toxicity, Extracellular polymeric substances, Metals, Copper inhibition

## Abstract

Pyritic minerals generally occur in nature together with other trace metals as impurities, that can be released during the ore oxidation. To investigate the role of such impurities, the presence of copper (Cu(II)), arsenic (As(III)) and nickel (Ni(II)) during pyrite mediated autotrophic denitrification has been explored in this study at 30 °C with a specialized microbial community of denitrifiers as inoculum. The three metal(loid)s were supplemented at an initial concentration of 2, 5, and 7.5 ppm and only Cu(II) had an inhibitory effect on the autotrophic denitrification. The presence of As(III) and Ni(II) enhanced the nitrate removal efficiency with autotrophic denitrification rates between 3.3 [7.5 ppm As(III)] and 1.6 [7.5 ppm Ni(II)] times faster than the experiment without any metal(loid) supplementation. The Cu(II) batches, instead, decreased the denitrification kinetics with 16, 40 and 28% compared to the no-metal(loid) control for the 2, 5 and 7.5 ppm incubations, respectively. The kinetic study revealed that autotrophic denitrification with pyrite as electron donor, also with Cu(II) and Ni(II) additions, fits better a zero-order model, while the As(III) incubation followed first-order kinetic. The investigation of the extracellular polymeric substances content and composition showed more abundance of proteins, fulvic and humic acids in the metal(loid) exposed biomass.

**Graphical Abstract:**

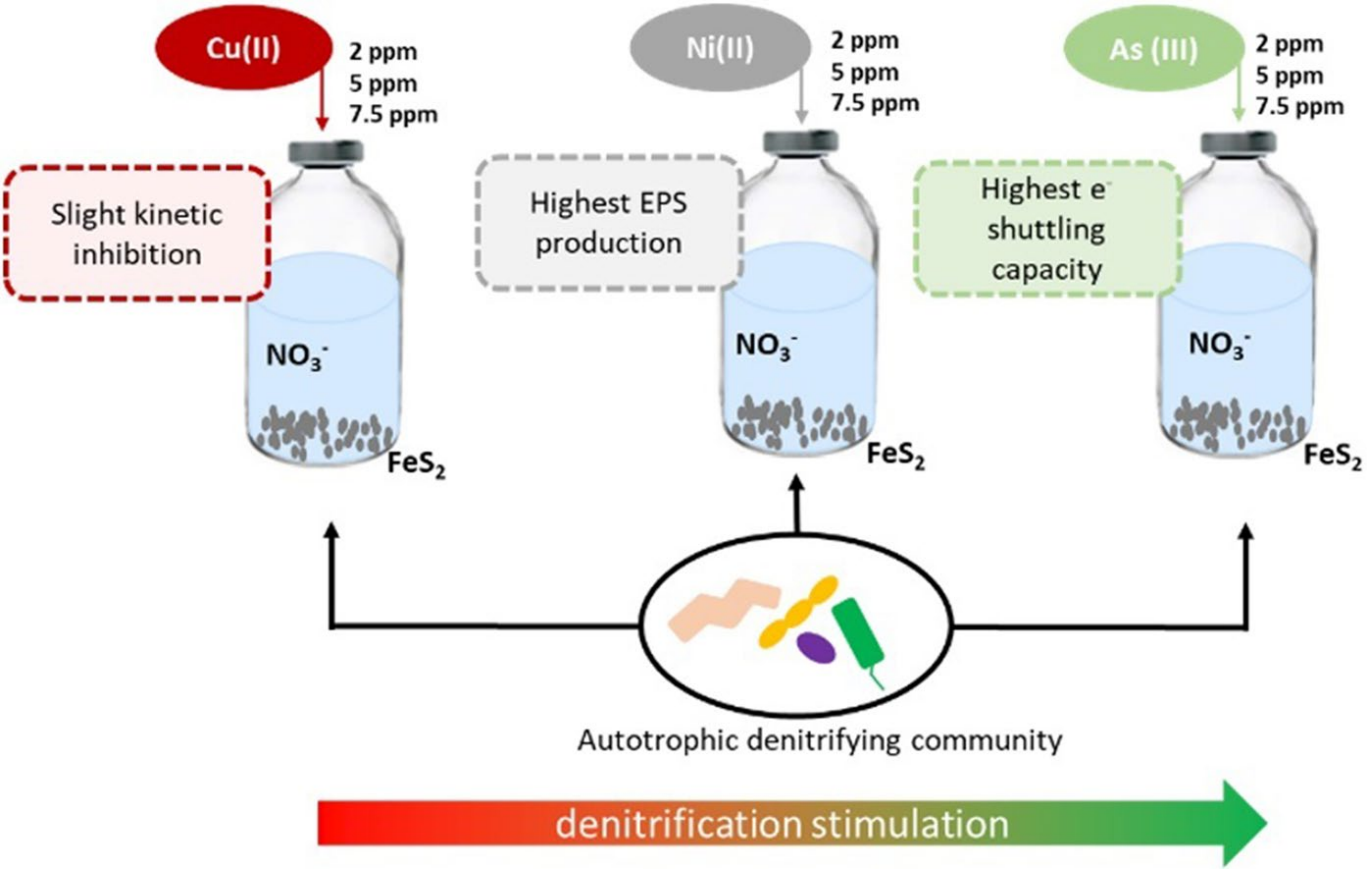

## Introduction

Pyrite (FeS_2_) oxidation in anoxic environments and autotrophic denitrification systems has gained more attention in the last two decades (Xu et al. 2019; Chen et al. 2020; Pang and Wang 2020). It is a natural process that occurs in pyrite rich aquifers (Zhang et al. 2012), in which the pyrite acts as electron donor for nitrate and nitrite conversion into gaseous nitrogen, and has been studied in laboratory experiments in order to better understand the mechanisms of this bioconversion. Pyrite is the most abundant sulfur-iron mineral in the earth’s crust (Di Capua et al. 2019) and has a low economic value since it is largely produced as waste material in mining and the construction sector (Bulut et al. 2014; Ferreira et al. 2021).

Natural FeS_2_ commonly contains copper (Cu) (chalcopyrite), arsenic (As) and nickel (Ni) impurities in different combinations depending on the geochemistry of the ore (Fleischer 1955; Abraitis et al. 2004; Savage et al. 2008). Pyrite oxidation can lead to the release of these heavy metals (HM) and metalloids in the water. Several species of autotrophic denitrifying bacteria have been found to use pyrite as electron donor and nitrate as electron acceptor, such as *Thiobacillus denitrificans*, *Sulfurimonas denitrificans*, *Thiobacillus thioparus*, *Ignavibacterium album* and also the genera *Parococcus* and *Thioprofundum* (Hu et al. 2020a; Carboni et al. 2021). Their denitrification activity can be either stimulated or inhibited (Igiri et al. 2018) by the presence of HMs. Magnesium (Mg), Ni, Cu, calcium (Ca), manganese (Mn), zinc (Zn), sodium (Na) are essential elements required in trace quantity for metabolic and redox functions (Igiri et al. 2018). On the other hand, metals such as aluminium (Al), lead (Pb), cadmium (Cd) and metalloids as As can be toxic to microorganisms even in low concentrations (Igiri et al. 2018).

Some studies reported the effects of HMs on autotrophic denitrification systems. Kiskira et al. (2018) tested Fe^2+^ mediated autotrophic denitrification in the presence of Cu, Ni and Zn with different microbial communities in batch assays. *Thiobacillus* dominated mixed cultures were in general more tolerant to the HMs tested, while the pure cultures were more affected with a maximum denitrification inhibition of 94% in the presence of 40 mg/L Cu or Zn. Moon et al. (2006) tested Zn, Cu and Chromium (Cr VI) on autotrophic denitrification performed with elemental sulfur as electron donor. Zn and Cu inhibited the process at concentrations as low as 0.5 ppm, while Cr(VI) did not affect the nitrate removal efficiency.

To the best of the authors' knowledge, there has not yet been a systematic study on the impact of Cu(II), Ni(II) and As(III) on pyrite driven autotrophic denitrification. Only Li et al. (2021a) investigated the simultaneous nitrate and arsenite removal in a pyrrhotite driven autotrophic denitrification system. Therefore, the present study assessed the influence of pyrite with the exogenous addition of Cu(II), Ni(II) and As(III) at 3 different concentrations (2, 5, and 7.5 ppm). The objective of this work was to investigate the effect of co-contaminants generally present as impurities in pyritic minerals on autotrophic denitrification in order to understand how the process can be affected by the presence of such metal(loid)s coming from the oxidation of the pyrite itself. This would allow to choose the more functional kind of ore (evaluating the impurity minerals present in it) for future autotrophic denitrification application. The kinetic parameters of pyrite driven autotrophic denitrification were determined and how the kinetics were affected by the presence of the metal(loid)s in solution was quantified to know if any inhibition or stimulation effect occurred. The effect of metal(loid) addition on the production of extracellular polymeric substances (EPS) was monitored to understand if the autotrophic denitrifying bacteria secreted such substances as protective barriers to respond to possibly toxic substances. Finally the electron shuttling capacity (ESC) of pyrite was investigated as well in the various conditions.

## Materials and methods

### Source of microorganisms

An enriched chemolithotrophic denitrifying culture was used in this study obtained from a fluidized bed reactor (FBR) operating for 220 days at mesophilic temperature (30 °C) for pyrite-driven autotrophic denitrification described in detail by Carboni et al. (2022). It was dominated by the genera *Pseudomonas*, *Azospira*, *Petrimonas* and *Lentimicrobium*. This microbial community was selected since it was already acclimatized to the same kind of mineral medium and used to the same process conditions applied in the present study.

### Mineral growth medium

The mineral medium was prepared according to Stams et al. (1993), briefly consisting of (g/L): 0.3 NaCl, 0.3 NH_4_Cl, 0.1 MgCl_2_·6H_2_O, 0.41 KH_2_PO_4_, 0.53 Na_2_HPO_4_·2H_2_O and 1 ml/L of each acid and alkaline trace elements solution. 0.11 g/L CaCl_2_·2H_2_O, 4 g/L NaHCO_3_, and 0.2 ml/L vitamin solutions were added filter-sterilized to the autoclaved medium. The pH of the medium was kept at 7–7.5. Nitrate was added as KNO_3_ to a final concentration of 200 mg NO_3_^−^/L. 5 g of FeS_2_ (99.8% grade, 45 µm diameter from Sigma-Aldrich (St. Louis, United States)) was added to every batch bottle. All chemicals used were of analytical grade (Fischer Scientific, Hampton, United States).

### Experimental design

The experiments were performed in 250 mL glass bottles with a working volume of 125 mL. Bottles were incubated at 30 °C and 150 rpm in a New Brunswick Scientific innova 44 incubator orbital shaker (Eppendorf, Hamburg, Germany). The anoxic medium and inocula (20% v/v) were added to the bottles, then sealed with butyl rubber stoppers (Ochs Laborbedarf, Bovenden, Germany) and aluminium crimp caps. The headspace of the bottles was flushed for 2 min with N_2_ gas to a final pressure of 1.5 atm. Cu(II), Ni(II) and As(III) were added separately from concentrated stock solutions of copper chloride (CuCl_2_), nickel chloride (NiCl_2_·6H_2_O) and sodium arsenite (Na_3_AsO_3_) in 2% KOH, to the final concentrations of 2, 5 and 7.5 mg/L in order to simulate a percentage present of such impurities in the pyrite between 0.005% and 0.02% and typical leaching of metals during aerobic and anoxic pyrite oxidation (Hu et al. 2020a, b).

The experiments were performed in triplicate. Controls without inoculum, pyrite or metals were analyzed in triplicate as well (Table 1) in order to investigate, respectively, any abiotic activity, the possible contribution of the metals on the denitrification activity and the denitrification in the absence of the added metal(loid)s.

**Table 1:** Experimental conditions of the batch experiments on the effect of Cu(II), Ni(II) and As(III) on pyrite-driven autotrophic denitrification (experiments were performed in triplicates)

Metals	Metal concentration [mg/L]	[NO_3_^−^]	[FeS_2_]	Inoculum	
Cu(II)	2	200 mg/L	5 g/bottle	20% (v/v)	Experiments
	5				
	7.5				
As(III)	2				
	5				
	7.5				
Ni(II)	2				
	5				
	7.5				
–	–		5 g/bottle	20% (v/v)	Controls
Cu(II)	2		–		
As(III)	2		–		
Ni(II)	2		–		
–	–		5 g/bottle	–	

### Kinetic analysis

A kinetic analysis was done to study the autotrophic denitrification in the presence of the Cu(II), Ni(II) and As(III). Zero, half and first order models were applied to fit the nitrate reduction process, the kinetic constant K, the correlation coefficient r^2^ and the empirical points were calculated. The analysis was done by fitting the zero-order (Eq. 1), half-order (Eq. 2) and first-order (Eq. 3) kinetic models, explained elsewhere (Wan et al. 2017; Li et al. 2021b), with the experimental points using Origin2018 software (OriginLab Corporation, Northampton, USA).

1 $${\text{C = C}}_{{0}} - {\text{K}}_{0} {\text{t}}$$

2 $${\text{C = }}\left( {{\text{C}}_{0}^{1/2} - 1/2{\text{K}}_{1/2} {\text{t}}} \right)^{2}$$

3 $${\text{C}} = {\text{C}}_{0} \exp \left( { - {\text{K}}_{1} {\text{t}}} \right)$$

where, C_0_ is the initial nitrate concentration (mg/L); t is the reaction time of nitrate removal (h); C is the nitrate concentration at a defined t (mg/L); K_0_ (mg/L h), K_1/2_ (mg^1/2^ / L^1/2^ h), and K_1_ (1/h) are the reaction constants of, respectively, zero-order, half-order and first order models.

### Statistical analysis

Statistical comparison of the data of residual nitrate at the end of the incubations from controls and each condition tested were compared by one-way analysis of variance (ANOVA) followed by the Tukey post hoc test (Lee and Lee 2018). The same statistical analysis was performed for the residual metal(oid)s. The analyses were performed with Minitab 17 Statistical Software (Minitab LCC, USA), where a difference marked with a p-value lower than 0.05 was considered statistically significant.

### Extracellular polymeric substances

Extracellular polymeric substances (EPS) are excreted by bacteria, and form a protective layer which can resist environmental toxic shocks as e.g. from metal(loid)s (Gonzalez et al. 2010). The different metal concentrations tested could alter the characteristics and components of the EPS (Luo et al. 2020). 3 g of solid samples for the EPS extraction and analysis were collected at the beginning and the end of the experiments and analysed as described by Luo et al. (2020). Loosely bounded EPS (LB-EPS) and tightly bounded EPS (TB-EPS) were analysed separately.

Solid samples were centrifuged at 2500×*g* for 15 min at 4 °C (Allegra x-30R centrifuge, Beckman Couiter, Brea, USA). The supernatant was collected for the LB-EPS fraction and the pellet was mixed with 10 mL of 0.05% NaCl solution. The samples were ultrasonicated at 150 W for 5 min (Bandelin sonorex digiplus, Berlin, Germany) and finally, centrifuged at 12,000×*g* for 20 min. The supernatant was then collected for the TB-EPS analysis.

3D Fluorescence Excitation-Emission Matrix (FEEM) was used to identify the EPS content (aromatic proteins, fulvic and humic acids and soluble microbial like products) on the prepared samples. A Shimadzu RF-6000 (Kyoto, Japan) was set to scan samples from 200 to 550 nm (excitation and emission wavelengths) at 6000 nm min^−1^ with an excitation and emission bandwidth of 3.0 nm and a voltage of 400 V for the photomultiplier tube (Costa et al. 2020). The EEM signals were corrected using the blank subtraction process (Murphy et al. 2010). The total organic carbon (TOC) of LB-EPS and TB-EPS samples was measured using a TOC analyzer (TOC-L, Shimadzu, Kyoto, Japan) and then, normalized to 10 mg/L.

### Electron shuttling capacity

The electron shuttling capacity (ESC) is a measure of the total electron accepting capacity of a material, in this case of the pyrite together with the metals. It was quantified following the methodologies reported by Covarrubias-García et al. (2020) and Valenzuela et al. (2017). 0.2 mL of sample was mixed with 0.2 mL of 0.5 M HCl solution and left standing for 30 min. At the same time, 0.2 mL of sample was reacted with the same volume of ferric citrate solution (20 mM) for 3 h. After the reaction with ferric citrate, 0.2 mL of the resuspended sample was left resting with the same volume of HCl solution for 30 min. After this, samples were centrifuged for 10 min at 10,000×*g* in an Eppendorf AG MiniSpin 5452 centrifuge (Eppendorf, Hamburg, Germany) and 0.2 mL of the supernatant was recovered and allowed to react for 10 min with 5 mL 0.2 g/L solution of 2,4,6-Tris(2-pyridyl)-1,3,5-triazine (ferrozine reagent). The ferrozine solution was buffered with 50 mM HEPES. The solution was then measured at 562 nm in a Shimadzu UV-1900 spectrophotometer (Shimadzu, Kyoto, Japan). All solutions used in this analysis were sparged with N_2_ for 30 min to ensure the absence of dissolved oxygen.

### Analytical methods

Liquid samples (1 mL) were taken daily from the batch bottles and filtered through a 0.22-mm membrane filter. pH was measured using a Mettler Toledo FiveEasy™ (FP20, United States) pH meter. Nitrite and nitrate concentrations were measured by ion chromatography (Dionex Aquion, Thermo Scientific, Waltham, United States) with an IonPac AS14A 4 × 250 mm column coupled with an AG14A 4 × 50 mm guard column and sodium carbonate 3.03 mM/sodium bicarbonate 0.97 mM eluent at a flow rate of 1 ml min^−1^ (Florentino et al. 2020).

Total Fe, Cu, As and Ni analyses were performed with an ICP-OES (ThermoFisher, Scientific, Waltham, United States) operated at RF power: 1.3 kW, argon plasma flow rate: 8 L min^−1^, auxiliary argon flow rate: 0.3 L min^−1^ and nebulizer argon flow rate: 0.8 L min^−1^ as described by (Costa et al. 2020). Fe, Cu, As and Ni were read on radial mode at, respectively, 238.204 nm, 327.395 nm, 188,980 nm and 231.604 nm.

## Results

### Pyrite based autotrophic denitrification performance

**Fig. 1:**
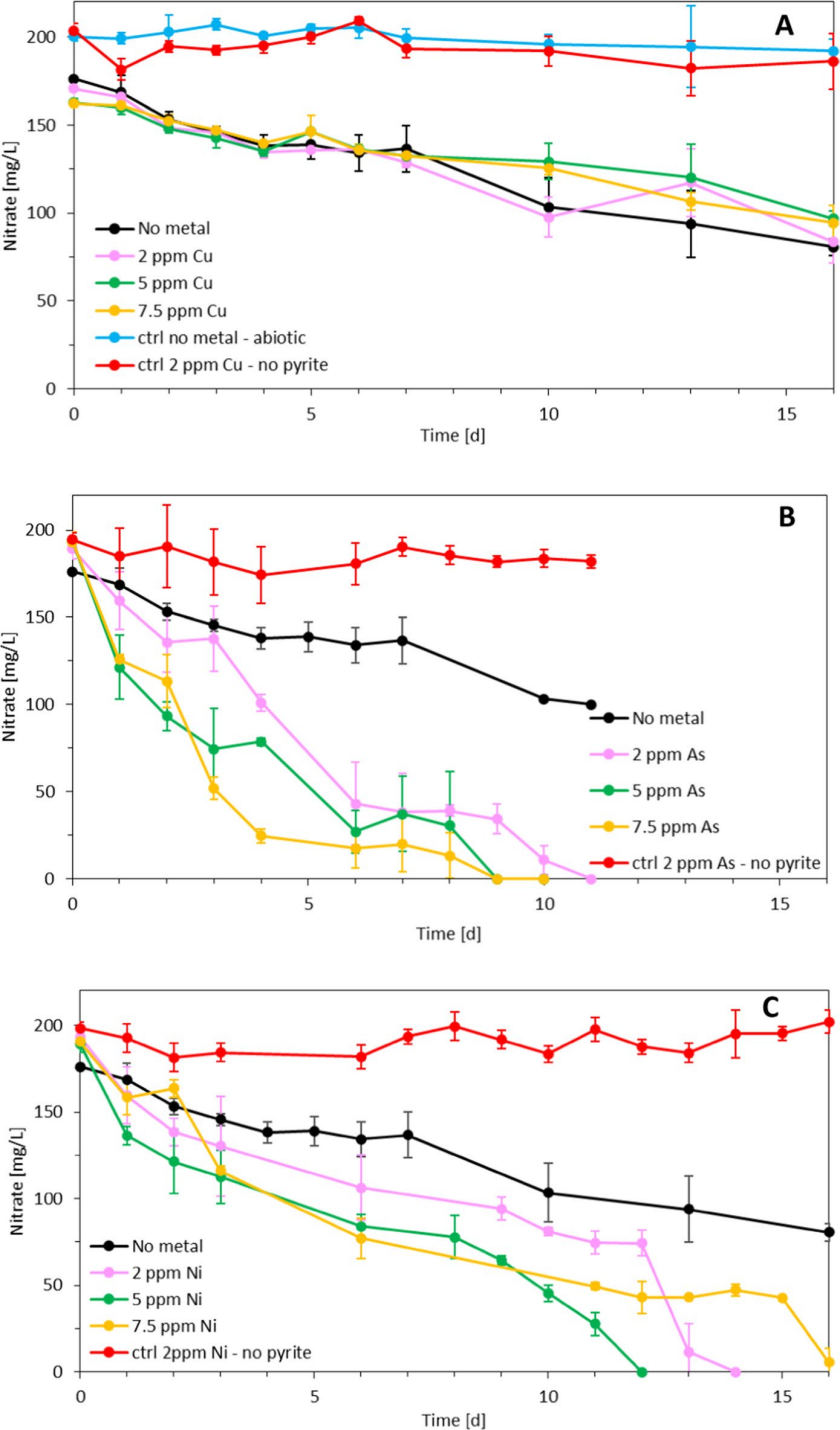
Evolution of nitrate concentration with time during pyrite-based autotrophic denitrification in presence of **A** Cu(II), **B** As(III) and **C** Ni(II)

Autotrophic denitrification coupled with pyrite oxidation was initially investigated in the absence of Cu, As and Ni (Fig. 1). In all triplicates no nitrite was detected as intermediate of denitrification and the pH remained stable in the range of 7.0 (± 0.4). During the 16 days of the experiment, the nitrate concentration decreased from 176.17 (± 0.46) to 80.61 (± 4.96) mg NO_3_^−^/L with a 54% nitrate removal efficiency. Figure 1 shows the nitrate evolution during the 16 days of the experiments in the bioassays with Cu(II) (Fig. 1A), As(III) (Fig. 1B) and Ni(II) (Fig. 1C) at different initial concentrations. The batch reactors with Cu(II) present no significant difference (p > 0.05) (Table 2) in comparison with the control where the denitrification occurred only with FeS_2_ in the absence of any metal. In particular, the experiments with 2 ppm of Cu(II) had a nitrate removal efficiency of 51% in 16 days, almost equal to that of the no-metal control, while in the experiments with 5 and 7.5 ppm of Cu(II) 40% and 41% denitrification was achieved, respectively. Figure 1A also reports the trend of nitrate in the abiotic control without metal and the one in the absence of the electron donor (pyrite) and 2 ppm of Cu(II), for which no denitrification was detected. This confirms that the denitrification was biological and that pyrite was exploited as electron donor while the metal did not have a role in the bioconversion.

**Table 2:** Tukey statistical comparison of residual nitrate (mg NO_3_^−^-N/L) residual metal(oid) (ppm) on the last day of the experiment

Experiment	Nitrate			Metal(oid)		
	Mean	SD	Statistical information^a^	Mean	SD	Statistical information^a^
No metal ctrl	18.2	1.12	B	–	–	–
Cu(II) 2 ppm	18.9	2.7	B	0	0	C
Cu(II) 5 ppm	21.8	0.9	B	0	0	C
Cu(II) 7.5 ppm	21.3	2.2	B	0	0	C
Cu(II) 2 ppm – No pyrite ctrl	42.1	3.5	A	0.14	0.08	C
As(III) 2 ppm	0	0	C	1.18	0	B
As(III) 5 ppm	0	0	C	2.39	0.54	A
As(III) 7.5 ppm	0	0	C	3.04	0.72	A
As(III) 2 ppm – No pyrite ctrl	41.1	0.9	A	0.91	0.04	BC
Ni(II) 2 ppm	0	0	C	0.18	0.03	C
Ni(II) 5 ppm	0	0	C	0.78	0.07	BC
Ni(II) 7.5 ppm	1.3	1.8	C	0.84	0	BC
Ni(II) 2 ppm – No pyrite ctrl	45.6	1.5	A	0.26	0.01	BC

^a^The same letter represents no significant differences (p > 0.05) with the compared condition

The incubations with As(III) instead, had a significant different behavior compared with the no-metal control (Fig. 1A, B). When As(III) was present at a concentration of 7.5 ppm, complete denitrification was achieved in 9 days, and after the first 4 days already 87% of the nitrate was reduced. Also the experiments with 5 ppm As(III) achieved complete denitrification in the first 9 days, while for the bottles in which there was only 2 ppm As(III), it took 11 days to completely reduce 190 mg NO_3_^−^/L. Also in this case, in the control without pyrite and with 2 ppm of As(III), no denitrification was measured, confirming that As(III) did not directly contribute to the denitrification process.

For all 3 Ni(II) concentrations (Fig. 1C) significantly improved denitrification activity was obtained compared to the no-metal experiment (p < 0.05). The fastest nitrate removal occurred in the 5 ppm experiment for which 100% NO_3_^−^ was removed in 12 days. The 2 ppm assay also reached complete denitrification in 14 days, being 2.3 times faster than the no-metal control. The 7.5 ppm Ni(II) assay instead reached 97% denitrification in 16 days, a residual nitrate concentration of 5.6 mg NO_3_^−^/L was measured at the end of the experiment.

According to the r^2^ values the no-metal control, Cu(II) and Ni(II) incubations follow the zero-order model, while the As(III) incubations follow the first-order kinetic model (Table 3). The K constant is an indicator of the reaction velocity. The zero-order kinetic indicates that the nitrate concentration is sufficient and does not limit the reaction rate, while the first-order kinetic indicates that the nitrate concentration is the main limiting factor of the reaction and if depleted the reaction stops. Table 3 shows Cu(II) negatively influenced the autotrophic denitrification since the kinetic constant in the Cu(II) experiments were 16, 40 and 28% lower than the no-metal control for the 2, 5 and 7.5 ppm incubations, respectively. On the other hand, As(III) and Ni(II) stimulated the nitrate removal since the K values are 3.3 and 1.6 times higher that of the no-metal control (Table 3). The K_0_ constant measured for the Ni(II) experiments was 12.25 L^−1^ h^−1^ in the 5 ppm, 11.29 L^−1^ h^−1^ in the 2 ppm and 9.47 L^−1^ h^−1^ in the 7.5 ppm incubation following the same trend of the ESC (Section “Influence of Cu(II), As(III) and Ni(II) on the ESC”).

**Table 3:** Kinetic parameters and correlation coefficients for the three kinetic models applied to nitrate removal by pyrite-based autotrophic denitrification in the presence of Cu(II), As(III) and Ni(II)

Experiment	Zero-order		Half-order		First-order	
	K_0_ [L^−1^ h^−1^]	r^2^	K_1/2_ [L^−1/2^ h^−1^]	r^2^	K_1_ [h^−1^]	r^2^
No metal	5.78	0.97	0.57	0.95	0.047	0.96
Cu(II) 2 ppm	4.86	0.88	0.49	0.87	0.040	0.90
Cu(II) 5 ppm	3.46	0.91	0.33	0.89	0.026	0.90
Cu(II) 7.5 ppm	4.15	0.98	0.36	0.97	0.032	0.97
As(III) 2 ppm	17.61	0.95	1.97	0.97	0.200	0.95
As(III) 5 ppm	17.06	0.86	2.91	0.87	0.279	0.95
As(III) 7.5 ppm	18.61	0.80	3.92	0.95	0.391	0.97
Ni(II) 2 ppm	11.29	0.77	1.01	0.71	0.088	0.67
Ni(II) 5 ppm	12.25	0.75	1.33	0.69	0.114	0.67
Ni(II) 7.5 ppm	9.47	0.77	0.92	0.73	0.082	0.71

### Evolution of Cu(II), As(III) and Ni(II) concentrations

**Fig. 2:**
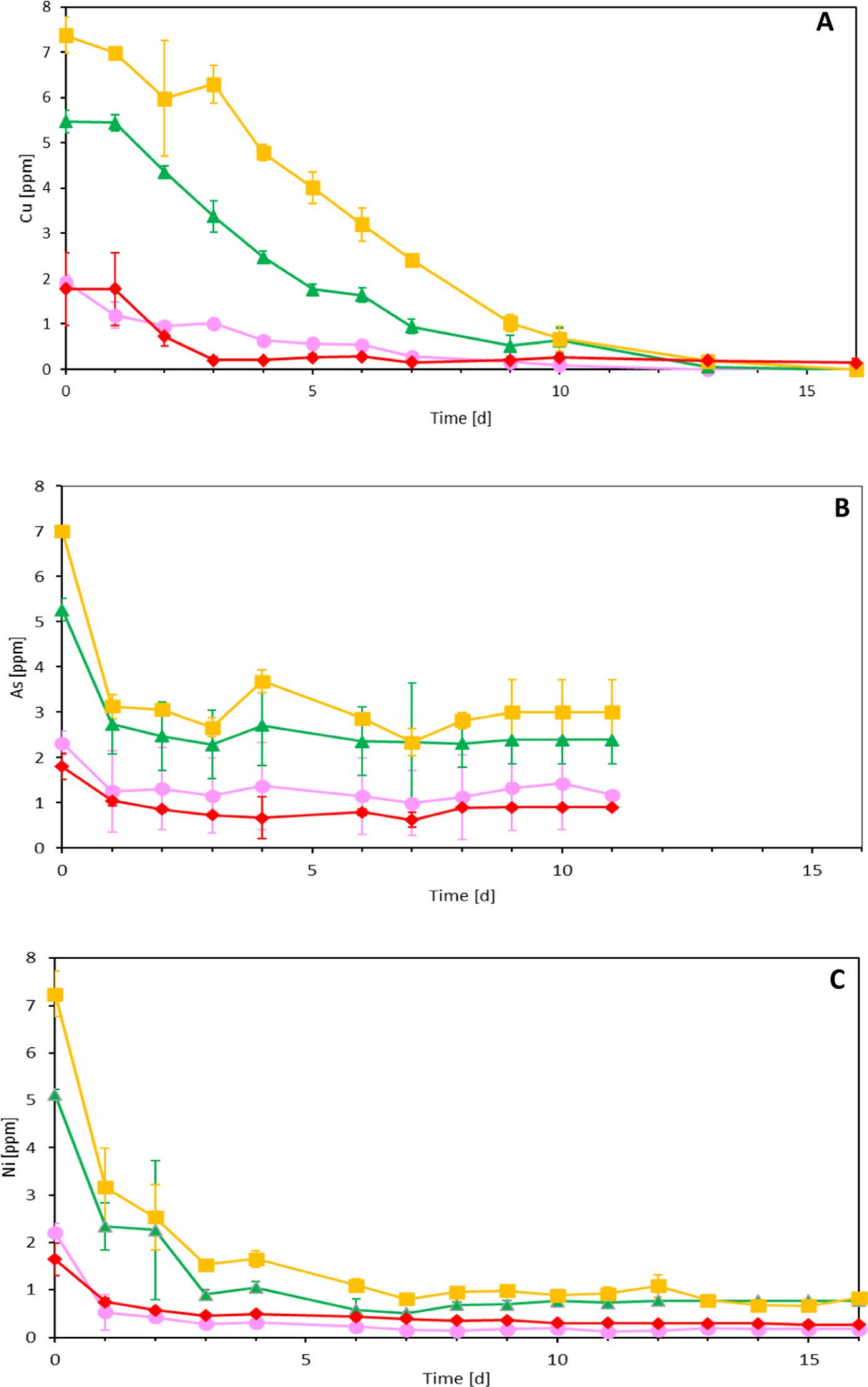
Total soluble **A** Cu(II), **B** As(III), and **C** Ni(II) profiles during the pyrite-based autotrophic denitrification experiments in the 2 ppm (pink color filled circle), 5 ppm (green triangle), 7 ppm (yellow square) incubations and no-pyrite 2 ppm control (red colored diamond). (Color figure online)

Figure 2 shows the trend of the Cu(II), As(III) and Ni(II) concentrations in the batch bottles at different initial concentrations. The Cu(II) incubations are different from those with As(III) and Ni(II). 100% copper removal was achieved for all 3 concentrations of 2, 5 and 7.5 ppm, but the decrease was not as fast as measured for As(III) and Ni(II). In the 2 ppm Cu(II) assay, a complete removal was achieved by day 13, while for the 5 and 7.5 ppm batches it was reached only on the last day of the incubation (day 16). The control with no pyrite and Cu(II) in a concentration of 2 ppm was also monitored, where 91% of the metal was removed by the last day of the incubation.

In the arsenite assay, instead, a fast decrease in its concentration was detected in the first day, with 1.07 ppm, 2.53 ppm and 3.87 ppm of As(III) removed in the 2, 5 and 7.5 ppm As(III) incubations, respectively. After the first day, As(III) concentrations remained stable over time until the last day of the experiment (day 11), differently from the Cu(II) experiments. The control with no pyrite and 2 ppm of As(III) was not significantly different (Table 2) with the incubation with 2 ppm As(III) achieving an As(III) removal efficiency of 50% on day 11.

In the Ni(II) experiment (Fig. 2C), the metal removal trend was similar to that of As(III) but the fast removal lasted 3 days for the 7.5 and 5 ppm assays and only 1 day for the 2 ppm incubation. After that, also in this case there was a stable residual Ni(II) concentration until day 16 of 0.17, 0.75 and 0.84 ppm of Ni(II) in the 2 ppm, 5 ppm and 7.5 ppm incubations, respectively.

### Influence of Cu(II), As(III) and Ni(II) on the ESC

**Fig. 3:**
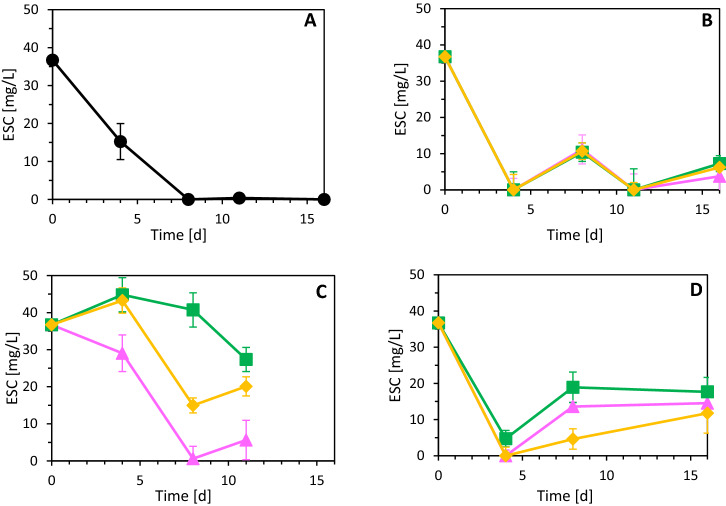
Evolution of electron shuttling capacity (ESC) of **A** no-metal control, **B** Cu(II), **C** As(III) and **D** Ni(II) experiments during pyrite-based autotrophic denitrification in the 2 ppm (pink triangle), 5 ppm (green square), and 7 ppm (yellow diamond) incubations. (Color figure online)

Figure 3 shows the ESC in the 4 conditions (no-metal, Cu(II), As(III) and Ni(II) experiments) and the 3 metal(loid) concentrations (2, 5 and 7.5 ppm). For the no-metal control (Fig. 3A), there is a gradual decrease in the ESC reaching 0 mg/L on day 8 of the experiment.

#### Cu(II) assays

In the Cu(II) experiment (Fig. 3b), the ESC reached 0 mg/L on days 4 for all the 3 concentration tested, but in the subsequent sampling points the values do not show a clear trend. The ESC behaviour for the Cu(II) incubations is similar for the 3 concentrations tested.

#### As(III) assays

In the As(III) incubations (Fig. 3C), the maximum ESC for the 3 tested concentrations was measured on day 4, and amounted to 30 mg/L, 44.8 mg/L and 43.25 mg/L for the 2, 5 and 7.5 ppm As(III) incubations, respectively. This peak is in line with the denitrification activity (Fig. 1B), where on day 4 already 47% [2 ppm As(III)], 59% [5 ppm As(III)] and 87% [7.5 ppm As(III)] of the initial nitrate was reduced. On day 8, the ESC of the 2 ppm As(III) incubation decreased to 0.6 mg/L and this correlated well with the denitrification activity between days 6 and 8 when only 4 mg NO_3_^−^/L were removed (Fig. 1B). The ESC in the 2 ppm As(III) experiment on day 11 increased up to 5.6 mg/L and in these last 3 days (8–11), the residual nitrate (40 mg/L) was completely reduced (Fig. 1B). The ESC trend of the 7.5 ppm As(III) incubation is higher, but similar to that of 2 ppm: the stationary phase in the nitrate removal (Fig. 1B) was measured from day 4–7 and, after that 100% denitrification was measured on day 9. For the 5 ppm As(III) incubation, on the other hand, after the peak on day 4 the ESC gradually decreased reaching 23.4 mg/L on day 11.

#### Ni(II) assays

In the Ni(II) assays (Fig. 3C), a clear drop in the ESC for the 3 metal concentrations tested was measured on day 4. After that, an increase until day 8 was measured up to 13.6 mg/L, 18.9 mg/L and 4.6 mg/L for the 2 ppm, 5 ppm and 7.5 ppm experiments, respectively. The ESC was then constant for the 2 and 5 ppm incubations, while it kept increasing up to 11.8 mg/L for the 7.5 ppm incubation. Therefore, on day 16 the ESC in the Ni(II) experiments was in the following order: 5 ppm > 2 ppm > 7.5 ppm, which matched with the trend of the denitrification performance (Fig. 1C).

### Influence of Cu(II), As(III) and Ni(II) on EPS

**Fig. 4:**
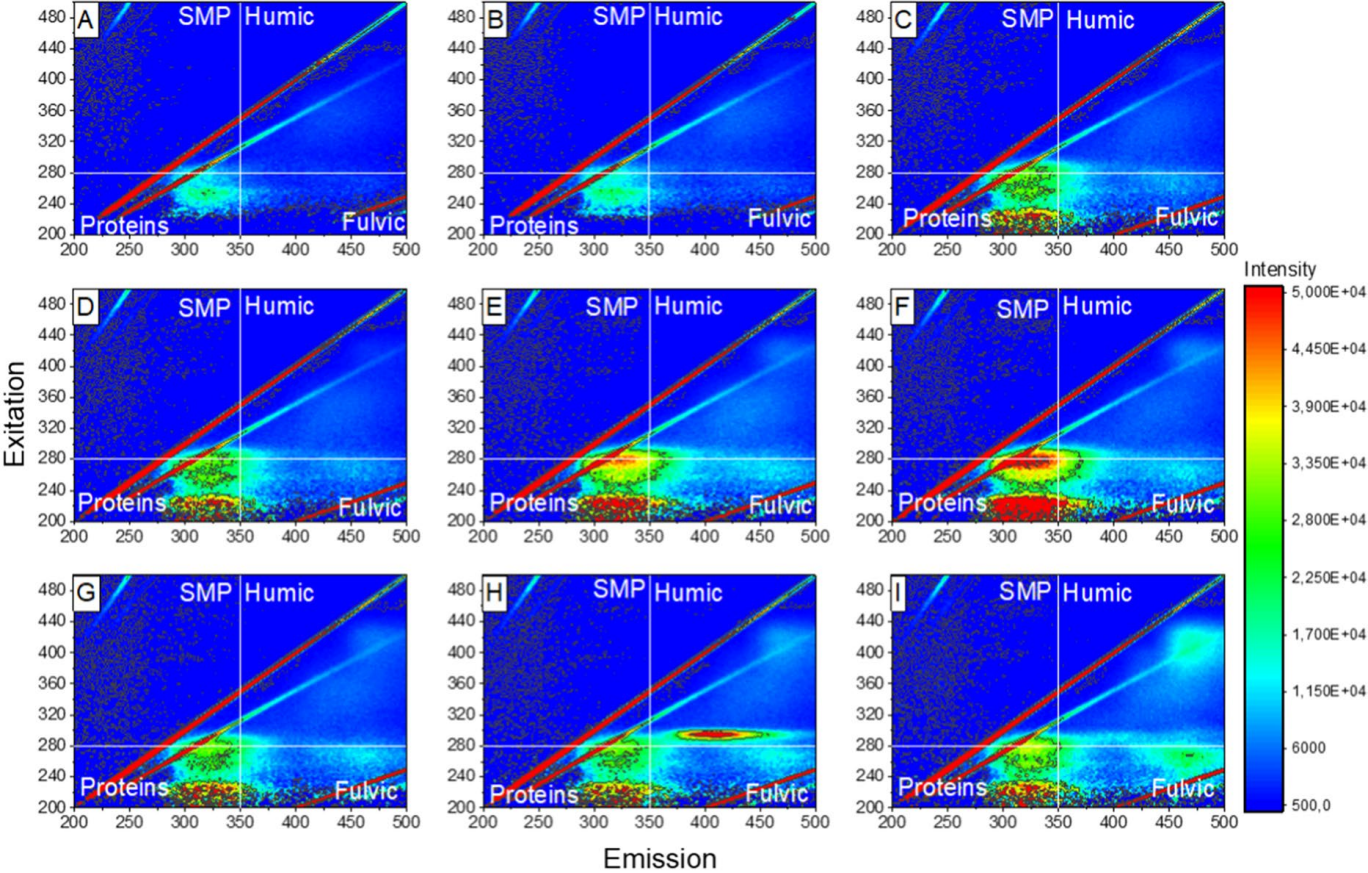
3D Fluorescence Excitation Emission Matrix (FEEM) spectra of Cu(II) incubations at **A** 2 ppm, **B** 5 ppm, **C** 7.5 ppm; As(III) incubations at **D** 2 ppm, **E** 5 ppm, **F** 7.5 ppm and Ni(II) incubations at **G** 2 ppm, **H** 5 ppm, **I** 7.5 ppm. The color scale corresponds to the intensity of the peaks, which correlates to their concentration, where red/orange correspond to the highest intensity and blue corresponds to the lowest intensity. (Color figure online)

In all cases, the LB-EPS (Fig. 4) showed a higher intensity than the TB-EPS (data not shown), likely because suspended biomass was applied in the batch experiments. For all the metal(loid)s tested, there is a clear increment in the EEM intensity from the 2 ppm to the 7.5 ppm incubations. Particularly, for the Cu(II) and Ni(II) incubations, there are higher peaks for the humic and fulvic acids-like materials, while for the As(III) incubation the main differences are in the protein and humic acids area of the matrix.

## Discussion

### Influence of As(III) on autotrophic denitrification

This study showed that low concentrations (2–7.5 ppm) of As(III) stimulated the nitrate removal efficiency by pyrite based autotrophic denitrification. This can be, most likely, attributed to the presence of the *Pseudomonas* genus in the inoculum (Carboni et al. 2022). It is an autotrophic denitrifier able to use sulfide and ferrous iron as electron donor (Chen et al. 2013; Su et al. 2015; Kiskira et al. 2017) and it is able to tolerate the presence of arsenic in the environment (Cai et al. 2009; Li et al. 2014). Li et al. (2021a) investigated the simultaneous arsenite and nitrate removal on a pyrrhotite (Fe_1−x_S, x = 0–0.125) based autotrophic denitrification system. As(III) in concentrations from 10 to 70 mg/L were tested and the denitrification activity decreased with the As(III) concentration, even if the bacteria were still able to perform nitrate removal at the maximum As(III) concentration supplied. A pathway in which the *Thiobacillus* genus was able to reduce NO_3_^−^ to N_2,_ while oxidizing FeS to SO_4_^2−^ and Fe^3+^ was proposed. At the same time the genus *Thiomonas* was oxidizing As^3+^ to As^5+^ and the latter was then precipitating as FeAsO_4_. Also the genus *Pseudomonas* is able to oxidize As^3+^ to As^5+^ (Paul et al. 2014). However, in order to confirm the pathway proposed by Li et al. (2021a), a more in depth study of the solid particles precipitated in the As(III) incubations is required, e.g. using FTIR or X-ray absorption spectroscopy or fluorescence in situ hybridization (FISH) can provide insights on metal speciation and the identification of the interactions between the metals and the microorganisms involved (Li et al. 2019). ^15^N isotope labelling of NO_3_^−^ would allow to determine the nitrate transformations and follow the formation of intermediates of the denitrification process (Hu et al. 2020b).

### Influence of Ni(II) on autotrophic denitrification

For all the 3 Ni(II) concentrations tested, a stimulative effect on the denitrification performance was observed and in particular the kinetic constant K_0_ was 1.90, 2.11, and 1.64 times higher than the K_0_ of the no-metal control for the 2 ppm Ni(II), 5 ppm Ni(II), and 7.5 ppm Ni(II) experiments, respectively (Table 3). Therefore, the best performance was observed in the 5 ppm Ni(II) experiment, suggesting that the 2 ppm and the 7.5 ppm trials were suboptimal concentrations to stimulate the denitrification. This is in line with Gikas (2008), who reported that 5 mg Ni/L improved the denitrification activity of activated sludges, while concentrations exceeding 10 mg Ni/L can inhibit the process. For all the three metal(oid)s tested their uptake (Fig. 2) did not directly impact the denitrification trends (Fig. 1). Ni(II), As(III) and Cu(II) did not act as electron donors for nitrate reduction since in the control experiments without pyrite no denitrification was detected (Fig. 1). Moreover, in all the three controls without pyrite the metals concentration decreased over time in the same way of the experiments done in the presence of the electron donor. This suggests that the consumption of the metal(oid)s in solution did not involve (or at least not completely) adsorption phenomenon on the mineral. This lead to the insight that the metal(oid)s were used/adsorbed by the microorganisms present in the community and most likely, such a process in the case of Ni(II) and As(III) stimulated their denitrification activity.

The genera *Azospira* and *Pseudomonas*—present in the inoculum (Carboni et al. 2022)—were found to be tolerant to the presence of Ni(II) in the solution (Paul et al. 2014; Zou et al. 2015). For *Pseudomonas* sp., nickel was shown to be an essential element for its chemolithotrophic growth (Gikas 2008). *Azospira* is a denitrifier able to use organic compounds as well as ferrous iron as electron donor. Zou et al. (2015) investigated heterotrophic denitrification in a FBR in the presence of Ni(II) and found that the microbial community tested, in which also *Azospira* was present, was partially inhibited at Ni(II) concentrations of 60 mg/L for 34 days, and then it was able to recover its denitrification activity. In the inoculum community used in the present study, the *Azospira* genus was present with a relative abundance of 5% (Carboni et al. 2022). But opposite to Zou et al. (2015), no acute inhibition by Ni(II) on the denitrification performance was detected (Fig. 1C).

### Influence of Cu(II) on autotrophic denitrification

The effect of Cu(II) on autotrophic denitrification was not remarkable, but induced an inhibitory effect (for all the 3 concentrations tested) in comparison with the no metal control. Despite being an essential element for the microbial growth (Gikas 2008), in the present study its impact on the denitrification activity was negative (Table 3). This is in line with Principi et al. (2006) and Ochoa-Herrera et al. (2018), who found that denitrifying bacteria are very sensitive to the presence of copper and the latter was the most bioaccumulating metal in municipal wastewater activated sludge among Cu(II), Ni(II) and Zn(II). Similarly, in the present study, Cu(II) was the only metal for which 100% of its presence in solution was removed during the experiment (Fig. 2A), while for the Ni(II) and As(III) experiments, there was always a residual concentration present in the liquid phase (Fig. 2).

## Conclusions

According to the results of the present study the application of pyritic minerals that might leach during the process would not hamper significantly the autotrophic denitrification reaction suggesting that the use of natural pyrite as electron donor is feasible. The presence of Ni(II) and As(III) stimulated the reduction of nitrate occurring between 3.3 and 1.6 times faster that of the no-metal control. On the contrary, the presence of Cu(II) inhibited the reaction since the kinetic constant in the 2, 5 and 7.5 ppm incubations were 16, 40 and 28%, respectively, lower than the no-metal control. For these reasons from an applicative point of view it is suggested to analyse the chemical composition of the pyritic mineral prior to start the wastewater autotrophic denitrification process. With the results obtained from this study, an ore without Cu(II) impurities is the one preferred to avoid inhibition of the denitrification process due to its presence in solution. The EPS study indicated that the microorganisms developed a clear increment in the EEM signal with the increasing concentration of metal(loid) investigated.

## Data Availability

The authors confirm that the data supporting the findings of this study are available within the article.

## References

[CR1] Abraitis PK, Pattrick RAD, Vaughan DJ (2004). Variations in the compositional textural and electrical properties of natural pyrite: a review. Int J Miner Process.

[CR2] Bulut G, Ünzile Y, Emrecan E, Ayhan AS (2014). Arsenic removal from aqueous solution using pyrite. J Clean Prod.

[CR3] Cai L, Guanghui L, Rensing C, Wang G (2009). Genes involved in arsenic transformation and resistance associated with different levels of arsenic-contaminated soils. BMC Microbiol.

[CR4] Carboni MF, Florentino AP, Costa RB, Zhan X, Lens PNL (2021). Enrichment of autotrophic denitrifiers from anaerobic sludge using sulfurous electron donors. Front Microbiol.

[CR5] Carboni MF, Mills S, Arriaga S, Collins G, Ijaz UZ, Lens PNL (2022). Autotrophic denitrification of high-nitrate wastewater in fluidized bed reactor using pyrite and elemental sulfur as electron donors—submitted in water research. SSRN Electron J.

[CR6] Chen C, Kuo LH, Fa CL, Mini H, Aijie W, Nanqi R, Duu JL (2013). Autotrophic and heterotrophic denitrification by a newly isolated strain *Pseudomonas Sp. C27*. Biores Technol.

[CR7] Chen Y, Zhiyu S, Zheng K, Li G, Junhua F, Chai H (2020). Study of pyrite based autotrophic denitrification system for low-carbon source stormwater treatment. J Water Process Eng.

[CR8] Costa RB, Bevilaqua D, Lens PNL (2020). Pre-treatment and temperature effects on the use of slow release electron donor for biological sulfate reduction. J Environ Manag.

[CR9] Covarrubias-García I, Quijano G, Aitor A, Sánchez-García JL, Rodríguez-López JL, Arriaga S (2020). Reduced graphene oxide decorated with magnetite nanoparticles enhance biomethane enrichment. J Hazard Mater.

[CR10] Di Capua F, Pirozzi F, Lens PNL, Esposito G (2019). Electron donors for autotrophic denitrification. Chem Eng J.

[CR11] Ferreira LP, Müller TG, Maykon C, De Oliveira CM, Peterson M (2021). Valorization of waste from coal mining pyrite beneficiation. J Environ Chem Eng.

[CR12] Fleischer, M. 1955. Minor elements in some sulfide minerals, fiftieth anniversary volume: 1905–1955, Alan M. Bateman. 10.5382/AV50.24.

[CR13] Florentino AP, Costa RB, Hu Y, Flaherty O, Lens PNL (2020). Long chain fatty acid degradation coupled to biological sulfidogenesis: a prospect for enhanced metal recovery. Front Bioeng Biotechnol.

[CR14] Gikas P (2008). Single and combined effects of nickel (Ni(II)) and cobalt (Co(II)) ions on activated sludge and on other aerobic microorganisms: a review. J Hazard Mater.

[CR15] González AG, Shirokova LS, Pokrovsky OS, Emnova EE, Martínez RE, Santana-Casiano JM, González-Dávila M, Pokrovski GS (2010). Adsorption of copper on *Pseudomonas aureofaciens*: protective role of surface exopolysaccharides. J Colloid Interface Sci.

[CR16] Hu Y, Guangxue W, Ruihua L, Xiao L, Zhan X (2020). Iron sulphides mediated autotrophic denitrification: an emerging bioprocess for nitrate pollution mitigation and sustainable wastewater treatment. Water Res.

[CR17] Hu Y, Jin Z, Hu Q, Hu J, Ni C, Li F (2020). Using stable isotopes to identify nitrogen transformations and estimate denitrification in a semi-constructed wetland. Sci Total Environ.

[CR18] Igiri BE, Okoduwa SIR, Idoko GO, Akabuogu EP, Adeyi OA, Ejiogu IK (2018). Toxicity and bioremediation of heavy metals contaminated ecosystem from Tannery wastewater: a review. J Toxicol.

[CR19] Kiskira K, Papirio S, van Hullebusch ED, Esposito G (2017). Fe(II)-mediated autotrophic denitrification: a new bioprocess for iron bioprecipitation/biorecovery and simultaneous treatment of nitrate-containing wastewaters. Int Biodeterior Biodegrad.

[CR20] Kiskira K, Papirio S, Fourdrin C, van Hullebusch ED, Esposito G (2018). Effect of Cu, Ni and Zn on Fe (II)-driven autotrophic denitrification. J Environ Manag.

[CR21] Lee S, Lee DK (2018). What is the proper way to apply the multiple comparison test? Korean. J Anesthesiol.

[CR22] Li C, Liu S, Ma T, Zheng M, Ni J (2019). Simultaneous nitrification, denitrification and phosphorus removal in a sequencing batch reactor (SBR) under low temperature. Chemosphere.

[CR23] Li R, Mengsha G, Wei W (2021). Simultaneous arsenite and nitrate removal from simulated groundwater based on pyrrhotite autotrophic denitrification. Water Res.

[CR24] Li H, Yaofeng L, Jianbo G, Yuanyuan S, Yanan H, Caicai L, Yi H, Xiaofeng S, Bowen L (2021). Effect of calcinated pyrite on simultaneous ammonia, nitrate and phosphorus removal in the BAF system and the Fe^2+^ regulatory mechanisms: electron transfer and biofilm properties. Environ Res.

[CR25] LiHuaming Y, Chunbo H (2014). Arsenic release from shallow aquifers of the Hetao Basin, inner Mongolia: evidence from bacterial community in aquifer sediments and groundwater. Ecotoxicology.

[CR26] Luo X, Hao Z, Qi L, Zhang J (2020). Effects of static magnetic field on *Chlorella Vulgaris*: growth and extracellular polysaccharide (EPS) production. J Appl Phycol.

[CR27] Moon HS, Sun WC, Kyoungphile N, Jaewan C, Jae YK (2006). Effect of reactive media composition and co-contaminants on sulfur-based autotrophic denitrification. Environ Pollut.

[CR28] Murphy KR, Kenna DB, Spencer RGM, Stedmon CA, Boehme JR, Aiken GR (2010). Measurement of dissolved organic matter fluorescence in aquatic environments: an interlaboratory comparison. Environ Sci Technol.

[CR29] Ochoa-Herrera V, León G, Banihani Q, Field JA, Sierra-Alvarez R (2018). Toxicity of copper(II) ions to microorganisms in biological wastewater treatment systems. Sci Total Environ.

[CR30] Pang Y, Wang J (2020). Insight into the mechanism of chemoautotrophic denitrification using pyrite (FeS_2_) as electron donor. Bioresour Technol.

[CR31] Paul D, Soumya P, Pinaki S (2014). Characterization of arsenite-oxidizing bacteria isolated from arsenic-contaminated groundwater of West Bengal. J Environ Sci Health—A Toxic/hazard Subst Environ Eng.

[CR32] Principi P, Villa F, Bernasconi M, Zanardini E (2006). Metal toxicity in municipal wastewater activated sludge investigated by multivariate analysis and in situ hybridization. Water Res.

[CR33] Savage SK, Stefan D, Lehner WS (2008). Impurities and heterogeneity in pyrite: influences on electrical properties and oxidation products. Appl Geochem.

[CR34] Stams AJM, Van Dijk JB, Dijkema C, Plugge CM (1993). Growth of syntrophic propionate-oxidizing bacteria with fumarate in the absence of methanogenic bacteria. Appl Environ Microbiol.

[CR35] Su JF, Si CS, Ting LH, Fang M, Shao FY, Zhou ZM, Sheng CZ (2015). Anaerobic nitrate-dependent iron(II) oxidation by a novel autotrophic bacterium, *Pseudomonas* Sp SZF15. J Environ Chem Eng.

[CR36] Valenzuela EI, Prieto-Davó A, López-Lozano NE, Hernández-Eligio A, Vega-Alvarado L, Juárez K, García-González AS, López MG, Cervantes FJ (2017). Anaerobic methane oxidation driven by microbial reduction of natural organic matter in a tropical wetland. Appl Environ Microbiol.

[CR37] Wan D, Yongde L, Yiyi W, Hongjie W, Shuhu X (2017). Simultaneous bio-autotrophic reduction of perchlorate and nitrate in a sulfur packed bed reactor: kinetics and bacterial community structure. Water Res.

[CR38] Xu B, Liangsheng S, Hua Z, Kang W (2019). The performance of pyrite-based autotrophic denitrification column for permeable reactive barrier under natural environment. Bioresour Technol.

[CR39] Zhang YC, Slomp CP, Broers HP, Bostick B, Passier HF, Böttcher ME, Omoregie EO, Lloyd JR, Polya DA, Van Cappellen P (2012). Isotopic and microbiological signatures of pyrite-driven denitrification in a Sandy Aquifer. Chem Geol.

[CR40] Zou G, Papirio S, van Hullebusch ED, Puhakka JA (2015). Fluidized-bed denitrification of mining water tolerates high nickel concentrations. Bioresour Technol.

